# Clinical symptom profile of hospitalized COVID-19 Brazilian patients according to SARS-CoV-2 variants

**DOI:** 10.4178/epih.e2023079

**Published:** 2023-08-28

**Authors:** Natália Satchiko Hojo-Souza, Vander Luis de Souza Freitas, Daniel Ludovico Guidoni, Fernanda Sumika Hojo de Souza

**Affiliations:** 1 Laboratory of Immunopathology, René Rachou Institute, Oswaldo Cruz Foundation, Belo Horizonte, Brazil; 2 Department of Computing, Federal University of Ouro Preto, Ouro Preto, Brazil

**Keywords:** COVID-19, Symptom assessment, SARS-CoV-2 variants, Disease outbreaks, Brazil

## Abstract

**OBJECTIVES:**

The aim of this study was to investigate the prevalence of the main symptoms in Brazilian coronavirus disease 2019 (COVID-19) patients hospitalized during 4 distinct waves, based on their infection with different severe acute respiratory disease coronavirus 2 (SARS-CoV-2) variants.

**METHODS:**

This study included hospitalized patients who tested positive for SARS-CoV-2 during 15 weeks around the peak of each of 4 waves: W1, ancestral strain/B.1 lineage (May 31 to September 12, 2020); W2, Gamma/P.1 variant (January 31 to May 15, 2021); W3, Omicron variant (December 5, 2021 to March 19, 2022); and W4, BA.4/BA.5 subvariants (May 22 to September 3, 2022). Symptom data were extracted from the Brazilian Severe Acute Respiratory Syndrome Database. Relative risks were calculated, and an analysis of symptom networks was performed.

**RESULTS:**

Patients who were hospitalized during the prevalence of the Gamma/P.1 variant demonstrated a higher risk, primarily for symptoms such as fatigue, abdominal pain, low oxygen saturation, and sore throat, than patients hospitalized during the first wave. Conversely, patients who were hospitalized during the predominance of the Omicron variant exhibited a lower relative risk, particularly for symptoms such as loss of smell, loss of taste, diarrhea, fever, respiratory distress, and dyspnea. Similar results were observed in COVID-19 patients who were hospitalized during the wave of the Omicron subvariants BA.4/BA.5. A symptom network analysis, conducted to explore co-occurrence patterns among different variants, revealed significant differential profiles across the 4 waves, with the most notable difference observed between the W2 and W4 networks.

**CONCLUSIONS:**

Overall, the relative risks and patterns of symptom co-occurrence associated with different SARS-CoV-2 variants may reflect disease severity.

## INTRODUCTION

Brazil has experienced 4 consecutive waves of coronavirus disease 2019 (COVID-19) over the nearly three-year span of the pandemic, which began in 2020. The first wave was characterized by cases and deaths due to the ancestral severe acute respiratory syndrome coronavirus 2 (SARS-CoV-2) B.1 lineage. Subsequent waves were marked by the predominance of variants/subvariants of concern (VOCs). The Gamma/P.1 (B.1.1.28.1) variant, which emerged in Brazil’s Amazonas State, caused the second wave [1,2]. The third wave was driven by the Omicron (B.1.1.529) VOC, which originated in South Africa [3], while the fourth wave was due to the prevalence of the Omicron BA.4/BA.5 subvariants [4]. Each of these variants, which carry numerous mutations primarily in the spike protein, has led to variations in spread, transmissibility, infectivity, and immune evasion, among other factors [5,6]. These characteristics have significantly impacted the clinical-epidemiological aspects of the disease, influencing symptoms, disease severity, hospitalization requirements, and fatality rates. Studying these aspects is crucial for public health, as they can inform managerial decision-making and guide the public on preventive measures. Unlike many countries where the Delta variant was prevalent in 2021, Brazil saw a predominance of the Gamma/P.1 variant. Most comparative studies on signs/symptoms have focused on the Beta, Delta, and Omicron variants [7,8]; thus, comparative data on symptoms caused by the Gamma/P.1 variant are lacking.

In this study, we examined the primary symptoms associated with SARS-CoV-2 variants in hospitalized COVID-19 patients and compared them to those exhibited by patients infected with the ancestral SARS-CoV-2. We gathered data from patients hospitalized during the peak of cases (15 weeks) in each wave and assessed the relative risk for each of the 12 symptoms. Additionally, we conducted a network analysis of symptom co-occurrence for each wave, highlighting the differences between them. It is important to note that we evaluated symptoms recorded by healthcare professionals in patients hospitalized with COVID-19, which may be more representative.

## MATERIALS AND METHODS

### Data extraction

**Figure 1.**
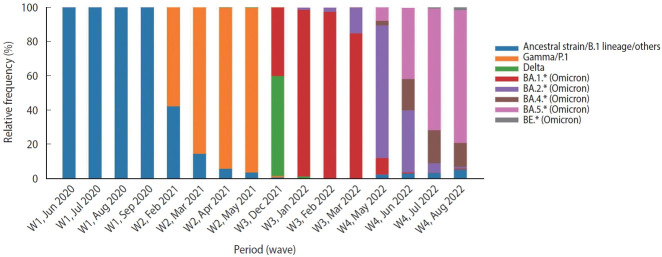
Relative frequency of SARS-CoV-2 variants during 4 COVID-19 waves in Brazil: ancestral lineage/B.1 (W1), Gamma/P.1 variant (W2), Omicron variant (W3), and Omicron BA.4/BA.5 subvariants (W4). Adapted from: https://www.genomahcov.fiocruz.br/dashboard-en/.

This study included data from hospitalized patients aged 18 years or older who tested positive for SARS-CoV-2 (using the quantitative reverse-transcriptase polymerase chain reaction technique [RT-PCR] or antigen test) during the 15 weeks around the peak of each of 4 waves, classified according to Brazil Fiocruz genomic surveillance data (https://www.genomahcov.fiocruz.br/dashboarden/): W1, ancestral strain/B.1 lineage (May 31 to September 12, 2020); W2, Gamma/P.1 variant (January 31 to May 15, 2021); W3, Omicron variant (December 5, 2021 to March 19, 2022); and W4, Omicron BA.4/BA.5 subvariants (May 22 to September 3, 2022). Figure 1 illustrates the relative frequency of SARS-CoV-2 variants during the study period. Symptom data, along with other demographic information such as age, sex, vaccine status, and outcome, were extracted from the Brazilian Severe Acute Respiratory Syndrome Database (SIVEP-Gripe; https://opendatasus.saude.gov.br/). This is a national and public database that contains de-identified data. The data was downloaded on September 12, 2022.

### Eligibility criteria

A total of 633,820 COVID-19 patients met the inclusion criteria. These patients were distributed heterogeneously across the 4 waves: W1 (n = 193,391), W2 (n = 339,234), W3 (n = 71,218), and W4 (n= 29,977). Patients were considered fully vaccinated if they had received a booster dose or completed the vaccine schedule (two doses for CoronaVac, Comirnaty, and Vaxzevria, or a single dose for Janssen) at least 14 days prior to the onset of symptoms. Patients with inconsistent vaccination data were excluded from the study.

### Statistical analysis

Frequency analysis (%) was conducted for categorical variables, while the median (interquartile range, IQR) was presented for continuous variables. The chi-square test for statistical significance [9] was employed to compare groups as needed. Poisson regression models with robust variance estimators [10] were utilized to calculate the risk of symptom manifestations between wave pairs. Relative risks (RRs) and 95% confidence intervals (CIs) were reported. The percentage of missing data for symptoms ranged from 10% to 40%; hence, the N values varied depending on the symptom.

A symptom network analysis was performed to explore co-occurrence patterns in each wave. Networks [11,12] were modeled as weighted graphs, with nodes representing symptoms and edges (links) representing the co-occurrence of a pair of symptoms in patients. Weights were associated with the nodes, indicating the number of patients manifesting such a symptom in that wave. Meanwhile, edge weights were related to the number of patients manifesting that pair of symptoms in each wave. All values were normalized by the highest value among the 4 waves, allowing the networks to be visually and topologically contrasted. In order to compare the differences, each network was represented as a 12× 12 matrix with the corresponding weights, had their differences computed between every possible pair of matrices, and their Frobenius norm was extracted. Higher values indicated a greater degree of distinctiveness. Vector norms are functions that consider an *A_mxn_* matrix as a vector of size *mn* allowing its association with a real number. In particular, the Frobenius norm is given as


$${||A||}_{p}={\left(\sum_{i=1}^{m}\;\sum_{j=1}^{n}\;{\left|a_{ij}\right|}^{p}\right)}^{1/p}\;,where\;p=2.$$

All analyses were performed using Python version 3.8.10 (Python Software Foundation, Beaverton, OR, USA) and the scipy statistical package (version 1.7.3), with a significance level of 0.05.

### Ethics statement

The data used in this study are publicly available and did not involve patients directly. It does not require an ethics committee’s approval.

## RESULTS

### Data of hospitalized coronavirus disease 2019 patients

The demographic data and vaccination status of hospitalized patients who tested positive for SARS-CoV-2 (RT-PCR or antigen test) included in the study in the 4 waves are shown in Table 1. Each wave spanned a 15-week interval, during which a significant reduction in COVID-19 patient hospitalizations was observed, particularly during the prevalence of the Omicron variant/subvariants (W3 and W4). However, a significant reduction in the in-hospital death rate was only noted in W4 (28.45%). In the first and second waves (W1 and W2), over 70% of hospitalized patients were aged between 40 years and 79 years. In contrast, during the third and fourth waves (W3 and W4), the majority of patients were aged 60 years or older. The vaccination status of patients varied across the waves. In W1, no hospitalized patients had been vaccinated, as COVID-19 vaccines were not yet available. By the second wave (W2), over 90% of hospitalized patients remained unvaccinated. Despite Brazil initiating its vaccination program on January 17, 2021, the process of acquiring vaccine lots was slow. Consequently, only a small percentage of the adult population had received their first dose by the peak of the Gamma variant wave. The situation markedly changed in W3, with 50% of hospitalized patients being fully vaccinated (having completed the vaccine regimen). This increased to over 60% in W4, with these patients having received a booster dose. The rise in hospitalizations among fully and booster-vaccinated patients during the prevalence of the Omicron variant/subvariants could be attributed to the variant’s potential to evade vaccine-induced immunity and/or the waning of immunity over time.

### Symptom prevalence

The most common symptoms among hospitalized COVID-19 patients, regardless of variant, included fever, cough, dyspnea, respiratory distress, and low oxygen saturation (SpO_2_). Less common symptoms encompassed sore throat, diarrhea, vomiting, abdominal pain, fatigue, loss of smell, and loss of taste (Table 2). However, the prevalence and nature of these symptoms have evolved over the course of the pandemic. The first wave was marked by a high prevalence of cough (78.52%), fever (69.11%), and respiratory disorders such as dyspnea (78.49%), respiratory distress (67.32), and SpO_2_ below 95% (69.46%). The second wave saw an even higher prevalence of dyspnea (83.20%), respiratory distress (72.13%), and SpO_2_ below 95% (81.19%). Unique symptoms at the onset of the pandemic included loss of taste (13.10% in W1 and 15.59% in W2) and loss of smell (13.15% in W1 and 15.24% in W2). Conversely, the third wave saw lower frequencies of fever (57.74%), cough (74.41%), dyspnea (73.74%), and respiratory distress (61.81%) compared to the first wave. Additionally, the third wave saw a significant decrease in the loss of taste (4.74%) and smell (4.23%). However, there was an increase in the prevalence of fatigue (32.12%) and abdominal pain (9.34%) compared to the first wave. Similar trends were observed during the fourth wave (Table 2).

### Relative risk of symptoms

**Figure 2.**
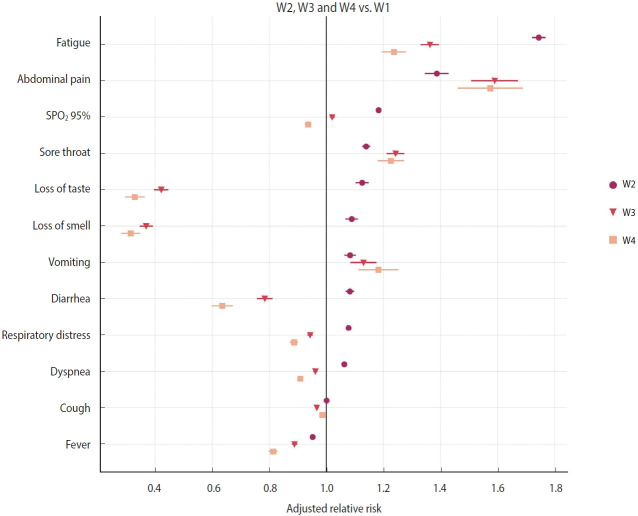
Adjusted relative risk for symptoms in coronavirus disease 2019 patients hospitalized during variant/subvariants predominance, relative to symptoms in patients hospitalized during the waves of the ancestral lineage/B.1 (W1), Gamma/P.1 variant (W2), Omicron variant (W3), and Omicron BA.4/BA.5 subvariants (W4). Error bars represent the 95% confidence interval. SpO_2_, oxygen saturation.

A relative risk analysis of symptoms was conducted, comparing W2 to W1, W3 to W1, and W4 to W1. The adjusted relative risk (aRR) for covariates such as age, sex, and vaccine status are depicted in Figure 2 and Table 2. During the period when the Gamma variant (W2) was dominant, COVID-19 patients who were hospitalized exhibited an increased risk primarily for symptoms such as fatigue (aRR, 1.74; 95% CI, 1.72 to 1.77; p< 0.001), abdominal pain (aRR, 1.39; 95% CI, 1.35 to 1.43; p< 0.001), SpO_2_ below 95% (aRR, 1.18; 95% CI, 1.18 to 1.19; p< 0.001), sore throat (aRR, 1.14; 95% CI, 1.13 to 1.15; p< 0.001), loss of taste (aRR, 1.13; 95% CI, 1.10 to 1.15; p< 0.001), loss of smell (aRR, 1.09; 95% CI, 1.07 to 1.11; p< 0.001), vomiting (aRR, 1.08; 95% CI, 1.06 to 1.10; p< 0.001), diarrhea (aRR, 1.08; 95% CI, 1.07 to 1.10; p< 0.001), respiratory distress (aRR, 1.08; 95% CI, 1.07 to 1.08; p< 0.001), and dyspnea (aRR, 1.06; 95% CI, 1.06 to 1.07; p< 0.001). These findings are in comparison to patients who were hospitalized during the first wave (W1) (Figure 2 and Table 2).

During the period of Omicron variant predominance, the main symptoms that exhibited a reduced relative risk in hospitalized patients included loss of smell (aRR, 0.37; 95% CI, 0.35 to 0.39; p< 0.001), loss of taste (aRR, 0.42; 95% CI, 0.40 to 0.45; p< 0.001), diarrhea (aRR, 0.78; 95% CI, 0.76 to 0.81; p< 0.001), fever (aRR, 0.89; 95% CI, 0.88 to 0.90; p< 0.001), respiratory distress (aRR, 0.94; 95% CI, 0.93 to 0.95; p< 0.001), dyspnea (aRR, 0.96; 95% CI, 0.95 to 0.97; p< 0.001), and cough (aRR, 0.97; 95% CI, 0.96 to 0.97; p< 0.001). Conversely, symptoms such as abdominal pain (aRR, 1.59; 95% CI, 1.51 to 1.67; p< 0.001), fatigue (aRR, 1.36; 95% CI, 1.33 to 1.40; p< 0.001), sore throat (aRR, 1.24; 95% CI, 1.21 to 1.27; p< 0.001), vomiting (aRR, 1.13; 95% CI, 1.09 to 1.18; p< 0.001), and SpO_2_ levels below 95% (aRR, 1.02; 95% CI, 1.01 to 1.03; p< 0.001) demonstrated an increased risk (Figure 2 and Table 2). These findings were consistent with the results observed in COVID-19 patients hospitalized during the wave of the Omicron BA.4/BA.5 subvariants (Figure 2 and Table 2).

### Vaccinated versus unvaccinated patients during the prevalence of the Omicron variant/subvariants

Patients hospitalized with COVID-19 during the Omicron waves (W3 and W4) were divided into vaccinated and unvaccinated groups to examine the potential influence of vaccination on symptom presentation. Given the assumption that vaccines may facilitate a more rapid clearance of the SARS-CoV-2 virus, a decrease in symptoms among vaccinated patients might be anticipated. However, our data revealed minimal significant differences in the prevalence of primary clinical symptoms between those vaccinated with at least one dose and unvaccinated hospitalized COVID-19 patients (Table 3). A minor reduction (1-4%) in the prevalence of symptoms such as fever, cough, dyspnea, respiratory distress, and vomiting was observed among vaccinated patients during the Omicron variant wave (W3). In the wave of Omicron subvariants (W4), only fever and respiratory distress demonstrated a lower prevalence among vaccinated patients.

### Networks of symptoms

**Figure 3.**
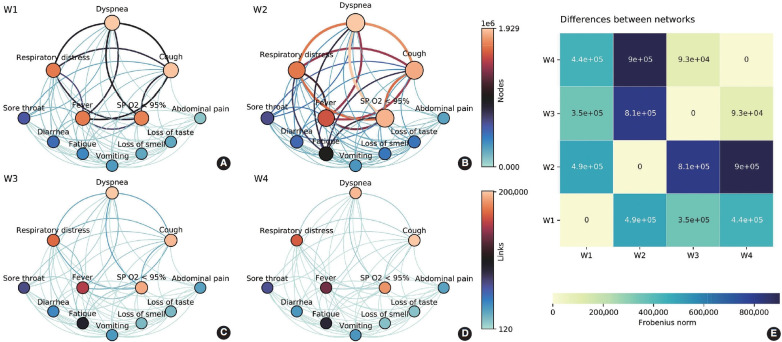
Network analysis of the 4 coronavirus disease 2019 waves in Brazil: ancestral lineage/B.1 (W1), Gamma/P.1 variant (W2), Omicron variant (W3) and Omicron BA.4/BA.5 subvariants (W4). (A-D) Symptom co-occurrence networks highlight symptoms’ relevance and interaction in each wave. (E) Pairwise comparison among networks, representing each wave and demonstrating the most similar/far-apart ones. SpO_2_, oxygen saturation.

Symptom co-occurrence networks of the 4 waves are shown in Figure 3A-D. The size of each node signifies the individual occurrence of symptoms, while the thickness and color of the edges illustrate the number of patients who exhibited both symptoms concurrently. The color of the nodes, on the other hand, represents strength, defined as the sum of all incident edges of the node. Despite utilizing the same color mapping for nodes and links, two color bars with distinct intervals were applied on the right side. Five nodes (dyspnea, respiratory distress, cough, fever, and SpO_2_ < 95%) were positioned at the top due to their relevance based on symptom occurrences. These symptoms demonstrated the highest occurrences and co-occurrences across the waves, particularly in the Gamma/P.1 wave (W2). It is noteworthy that fatigue, which was comparable to vomiting, loss of smell, and loss of taste in W1, gained significance in W2 (aRR, 1.74; Figure 2). This symptom had a higher number of co-occurrences with other relevant symptoms such as respiratory distress, dyspnea, cough, and SpO_2_ < 95%. The heatmap (Figure 3E) offers a pairwise comparison of the 4 networks. W3 and W4 are the most similar, while W2 and W4 are the most dissimilar, which aligns with the observed symptom manifestations across the 4 waves.

## DISCUSSION

The demographic data of hospitalized COVID-19 patients showed a different pattern during the waves of predominance of the Omicron variant and its subvariants. The hospitalization rate was lower during the peak period of cases compared to W1 and W2, potentially due to increased vaccine coverage or a lower intrinsic virulence of the Omicron variant and its subvariants [13]. The rise in the hospitalization rate of fully vaccinated and booster-vaccinated patients during the prevalence of the Omicron variant (W3) and BA.4/BA.5 subvariants (W4), respectively, could be attributed to waning immunity from vaccination [14,15] or evasion of vaccine-induced immunity [16,17]. Vaccination in Brazil commenced on January 17, 2021, initially targeting those over 60 years of age and healthcare workers, who primarily received two doses of CoronaVac (Sinovac/Butantan). Given that immunity tends to decrease after 4-6 months, it is plausible that the elderly were unprotected at the peak of wave W3. The high rate of hospitalization among patients who had received the booster dose during W4 also suggests waning immunity, as the administration of the booster vaccination (third dose) began on September 15, 2021, starting with the elderly. Consequently, at the peak of wave W4, the immunity of the elderly may have diminished. It is noteworthy that the hospitalization of COVID-19 patients aged 60 years and older at the peak of W3 and W4 was 70.03% and 76.27%, respectively. A prior study revealed that breakthrough infections occurred in a significant percentage, primarily among hospitalized patients aged 70 years or older, who were the first to be immunized with an inactivated virus-based vaccine [18]. Despite the relatively small sample size, a study conducted in the Northeast region of Brazil indicated that during the Omicron wave, there was a decrease in hospitalization and death rates compared to previous periods. However, fatal cases were predominantly among older individuals aged 60 or over, with 23.1% and 34.6% being booster and fully vaccinated, respectively [19].

Despite its name, SARS-CoV-2, it is now understood that COVID-19 is a systemic disease capable of causing complications beyond the lungs. The invasion of cells by SARS-CoV-2 is facilitated by the spike protein’s high affinity for angiotensin-converting enzyme 2 (ACE2). This enzyme serves as the virus receptor on the surface of the host cell and is expressed in a variety of tissues, including respiratory, gastrointestinal, nervous, cardiovascular, and renal tissues. This widespread expression could explain the diverse symptoms reported by COVID-19 patients [20].

According to the World Health Organization, the most prevalent symptoms of COVID-19 include fever, dry cough, and fatigue. Some patients may also experience less common symptoms such as loss of taste or smell, nasal congestion, sore throat, headache, muscle or joint pain, nausea, vomiting, and diarrhea, among others. Severe cases of COVID-19 may present with shortness of breath, loss of appetite, confusion, persistent chest pain or pressure, and a temperature exceeding 38°C [21]. However, preliminary reports suggest that the symptom patterns exhibited by COVID-19 patients have evolved throughout the course of the pandemic [8,22]. Initial studies indicated that the most frequently observed clinical symptoms in patients infected with the original Wuhan strain of SARS-CoV-2 were fever, cough, fatigue, and dyspnea, with diarrhea being relatively rare [23-25]. Our findings align with these early studies, as we observed a similar symptom pattern in patients hospitalized during the first wave of the pandemic. Notably, we found a high prevalence of patients experiencing respiratory distress (67.32%) and SpO_2_ levels below 95% (69.46%) (Table 2). Furthermore, we noted a significant increase in the frequency of fatigue, abdominal pain, and sore throat in patients hospitalized during subsequent waves (W2, W3, and W4). This suggests a shift in symptomatology corresponding to the emergence of different SARS-CoV-2 variants.

It can be observed that the majority of symptoms resulting from the Gamma variant infection are akin to those of the wild-type SARS-CoV-2, albeit with an intensification of respiratory tract symptoms. Another work utilized co-occurrence network analysis with long COVID patients to uncover intricate relationships among symptoms, revealing that abnormal breathing, chest/throat pain, and fatigue are notably interconnected [26]. In a similar vein, our analysis of symptom profiles and networks indicates a more severe COVID-19 manifestation in patients infected with the Gamma variant, characterized by pronounced co-occurrences of symptoms related to respiratory dysfunctions. The strong association with fatigue could be a result of these respiratory symptoms. The Gamma/P.1 variant has proven to be more infectious and lethal than the wild-type SARS-CoV-2, leading to an increase in hospitalizations, intensive care unit admissions, and the need for invasive mechanical ventilation, even among young adults [27,28]. At the height of the wave caused by the Gamma variant, when vaccines were not yet widely available, daily COVID-19 deaths exceeded 3,000-4,000 [29]. Factors such as diminishing immunity, immune evasion, and the Gamma/P.1 variant’s higher inherent transmissibility compared to the pre-existing lineage may have contributed to its resurgence and dominance in the state of Amazonas [1]. Accordingly, a genomic sequencing study combined with epidemiological data revealed that the Gamma/P.1 lineage acquired 17 mutations, including a trio in the spike protein (K417T, E484K, and N501Y) associated with increased binding to the human ACE2 receptor, making it an estimated 1.7-2.4 times more transmissible than the previous lineage [28].

Our results indicate that the characteristic symptoms of COVID-19 have evolved throughout the various waves instigated by SARS-CoV-2 variants. Notably, there was a significant decrease in chemosensory dysfunction (loss of taste and smell) during waves W3 and W4. This symptom was a distinct and valuable diagnostic marker at the onset of the pandemic. The prevalence of olfactory dysfunction caused by the Omicron variant/subvariants was around 3-4%, representing an approximate 4-fold decrease compared to previous variants. This aligns with a meta-analysis that pooled several studies from diverse populations [30].

Patients infected with the Omicron variant demonstrated a reduced risk of experiencing loss of smell (aRR, 0.37), loss of taste (aRR, 0.42), and diarrhea (aRR, 0.78). This was also observed in patients infected with the Omicron BA.4/BA.5 subvariants. Conversely, during the period of Omicron variant dominance, hospitalized patients exhibited an increased risk of abdominal pain (aRR, 1.59) and fatigue (aRR, 1.36). However, unlike the W2 variant, the symptom network analysis did not indicate increased co-occurrences, suggesting these symptoms are non-specific. This pattern was similarly observed in patients hospitalized during the prevalence of the Omicron BA.4/BA.5 subvariants.

A comparison of the prevalence of central clinical symptoms between vaccinated and unvaccinated patients infected with the Omicron variant revealed few significant differences. This suggests that patients who have been previously immunized may not be protected due to immune escape and/or waning immunity. Despite the limited data available, a 2021 study involving hospitalized COVID-19 patients found no significant differences in symptoms between those who were vaccinated and those who were not [31]. Another study indicated that vaccination status does not influence chemosensory dysfunction in individuals who have contracted the disease and reported symptoms via a telephone interview [32].

Altogether, our study adds new insights into the main symptoms caused by SARS-CoV-2 in hospitalized COVID-19 patients according to different variants. A comprehensive understanding of these clinical symptoms can aid in the effective medical management and appropriate treatment of these patients. The symptom networks observed during the predominance of the Omicron variant and its subvariants suggest that an early differential diagnosis for COVID-19 based on symptoms has become increasingly complex. This complexity arises from the diffuse nature of multiple symptoms that can easily be mistaken for those of other respiratory diseases.

The main strength of our study lies in its large sample size, which encompasses 633,820 hospitalized COVID-19 patients from various stages of the pandemic. Another significant feature is the method of symptom collection, which was conducted by healthcare professionals in a hospital setting, rather than relying on potentially subjective self-reported symptoms via telephone interviews. The data we analyzed were derived from mandatory hospital notifications and are accessible in a national, public database of deidentified data (SIVEP-Gripe database). Conversely, a key limitation of our study is the absence of information in the aforementioned database regarding non-hospitalized patients. The symptom prevalence in this group may differ, as they may experience mild or moderate symptoms that do not necessitate hospitalization. Consequently, we were unable to characterize the symptom profile of outpatient cases.

## Figures and Tables

**Table 1.** Data from hospitalized coronavirus disease 2019 patients stratified by period according to the predominance of variants^1^

Variables		W1 (n=193,391)	W2 (n=339,234)	W3 (n=71,218)	W4 (n=29,977)
In-hospital death		65,148 (33.69)	125,231 (36.92)	25,634 (35.99)	8,527 (28.45)
Age, median (IQR), yr		62 (36-88)	58 (37-79)	71 (37-105)	74 (38-110)
Age (yr)					
	18-39	24,365 (12.60)	49,312 (14.54)	7,816 (10.97)	2,663 (8.88)
	40-59	62,846 (32.50)	133,280 (39.29)	13,528 (19.00)	4,449 (14.84)
	60-79	76,635 (39.63)	126,891 (37.41)	28,662 (40.25)	11,854 (39.54)
	≥80	29,545 (15.28)	29,751 (8.77)	21,212 (29.78)	11,011 (36.73)
Sex					
	Female	85,217 (44.06)	151,835 (44.76)	34,739 (48.78)	15,606 (52.06)
	Male	108,174 (55.94)	187,399 (55.24)	36,479 (51.22)	14,371 (47.94)
Vaccine status					
	Booster	0 (0.00)	0 (0.00)	9,141 (12.84)	18,393 (61.36)
	Full	0 (0.00)	3,432 (1.01)	35,978 (50.52)	7,518 (25.08)
	Partial	0 (0.00)	24,248 (7.15)	3,580 (5.03)	1,049 (3.50)
	Unvaccinated	193,391 (100)	311,554 (91.84)	22,519 (31.62)	3,017 (10.06)

Values are presented as number (%). IQR, interquartile range.

1 W1, ancestral strain/B.1 lineage (2020/05/31 to 2020/09/12); W2, Gamma/P.1 variant (2021/01/31 to 2021/05/15); W3, Omicron variant (2021/12/05 to 2022/03/19); W4, Omicron BA.4/BA.5 subvariants (2022/05/22 to 2022/09/03).

**Table 2.** Symptom frequency (%) and aRR data in hospitalized coronavirus disease 2019 patients stratified by period according to predominance variants^1^

Symptoms	W1 (n=193,391)	W2 (n=339,234)	W3 (n=71,218)	W4 (n=29,977)	aRR (95% CI)^***^		
	Wuhan strain	Gamma/P.1	Omicron	BA.4/BA.5	W2 vs. W1	W3 vs. W1	W4 vs. W1
Fever	171,144	287,315	55,978	23,155	-	-	-
	118,271 (69.11)	191,091 (66.51)	32,324 (57.74)	11,710 (50.57)	0.95 (0.95, 0.96)	0.89 (0.88, 0.90)	0.81 (0.80, 0.83)
Cough	175,016	298,573	59,731	25,464	-	-	-
	137,419 (78.52)	235,838 (78.99)	44,448 (74.41)	19,315 (75.85)	1.00 (1.00, 1.00)	0.97(0.96, 0.97)	0.99 (0.98, 1.00)
Sore throat	147,800	243,637	48,488	20,805	-	-	-
	31,629 (21.40)	61,549 (25.26)	12,019 (24.79)	4,957 (23.83)	1.14 (1.13, 1.15)	1.24 (1.21, 1.27)	1.23 (1.18, 1.27)
Dyspnea	175,462	305,632	58,904	24,335	-	-	-
	137,721 (78.49)	254,278 (83.20)	43,433 (73.74)	17,002 (69.87)	1.06 (1.06, 1.07)	0.96 (0.95, 0.97)	0.91 (0.90, 0.92)
Respiratory distress	164,908	281,955	54,104	22,708	-	-	-
	111,010 (67.32)	203,361 (72.13)	33,443 (61.81)	13,184 (58.06)	1.08 (1.07, 1.08)	0.94 (0.93, 0.95)	0.89 (0.87, 0.90)
SpO_2_ <95%	166,874	296,577	57,561	23,854	-	-	-
	115,903 (69.46)	240,804 (81.19)	40,919 (71.09)	15,814 (66.29)	1.18 (1.18, 1.19)	1.02 (1.01, 1.03)	0.94 (0.92, 0.95)
Diarrhea	147,189	241,100	46,952	19,965	-	-	-
	26,930 (18.30)	48,610 (20.16)	6,300 (13.42)	2,160 (10.82)	1.08 (1.07, 1.10)	0.78 (0.76, 0.81)	0.64 (0.60, 0.67)
Vomiting	144,477	235,551	46,560	20,075	-	-	-
	15,580 (10.78)	28,062 (11.91)	5,421 (11.64)	2,381 (11.86)	1.08 (1.06, 1.10)	1.13 (1.09, 1.18)	1.18 (1.11, 1.25)
Abdominal pain	78,074	230,880	45,977	19,827	-	-	-
	4,886 (6.26)	20,654 (8.95)	4,296 (9.34)	1,802 (9.09)	1.39 (1.35, 1.43)	1.59 (1.51, 1.67)	1.57 (1.46, 1.69)
Fatigue	79,958	247,403	48,493	20,691	-	-	-
	18,650 (23.32)	101,233 (40.92)	15,575 (32.12)	6,031 (29.15)	1.74 (1.72, 1.77)	1.36 (1.33, 1.40)	1.24 (1.19, 1.28)
Loss of smell	78,548	234,501	45,252	19,479	-	-	-
	10,332 (13.15)	35,742 (15.24)	1,916 (4.23)	633 (3.25)	1.09 (1.07, 1.11)	0.37 (0.35, 0.39)	0.31 (0.28, 0.35)
Loss of taste	78,324	234,184	45,228	19,473	-	-	-
	10,261 (13.10)	36,512 (15.59)	2,145 (4.74)	649 (3.33)	1.13 (1.10, 1.15)	0.42 (0.40, 0.45)	0.33 (0.30, 0.36)

Values are presented as number or number (%). aRR, adjusted relative risk; CI, confidence interval; SpO_2_, oxygen saturation.

1 W1, ancestral strain/B.1 lineage (2020/05/31 to 2020/09/12); W2, Gamma/P.1 variant (2021/01/31 to 2021/05/15); W3, Omicron variant (2021/12/05 to 2022/03/19); W4, Omicron BA.4/BA.5 subvariants (2022/05/22 to 2022/09/03).

*** p<0.001.

**Table 3.** Symptom frequency (%) in vaccinated and unvaccinated coronavirus disease 2019 patients hospitalized during the prevalence of the Omicron variant (W3) and BA.4/BA.5 subvariants (W4)

Symptom	W3			W4		
	Vaccinated	Unvaccinated	p-value	Vaccinated	Unvaccinated	p-value
Fever	56.19 (20,298/36,122)	60.57 (12,026/19,856)	<0.001	50.12 (10,014/19,982)	53.45 (1,696/3,173)	<0.001
Cough	73.92 (28,454/38,495)	75.32 (15,994/21,236)	<0.001	76.04 (16,747/22,024)	74.65 (2,568/3,440)	0.080
Sore throat	24.35 (7,738/31,780)	25.62 (4,281/16,708)	0.002	23.98 (4,322/18,026)	22.85 (635/2,779)	0.203
Dyspnea	72.89 (27,695/37,998)	75.28 (15,738/20,906)	<0.001	69.59 (14,623/21,014)	71.64 (2,379/3,321)	0.018
Respiratory distress	61.12 (21,489/35,160)	63.10 (11,954/18,944)	<0.001	57.49 (11,290/19,639)	61.71 (1,894/3,069)	<0.001
SpO_2_ < 95%	71.02 (26,446/37,235)	71.20 (14,473/20,326)	0.649	66.18 (13,663/20,646)	67.05 (2,151/3,208)	0.340
Diarrhea	13.18 (4,067/30,867)	13.88 (2,233/16,085)	0.034	10.64 (1,840/17,294)	11.98 (320/2,671)	0.041
Vomiting	11.23 (3,432/30,574)	12.44 (1,989/15,986)	<0.001	11.84 (2,060/17,404)	12.02 (321/2,671)	0.812
Abdominal pain	9.04 (2,741/30,307)	9.92 (1,555/15,670)	0.002	8.96 (1,540/17,189)	9.93 (262/2,638)	0.114
Fatigue	32.67 (10,439/31,949)	31.04 (5,136/16,544)	<0.001	29.01 (5,197/17,916)	30.05 (834/2,775)	0.269
Loss of smell	4.13 (1,231/29,838)	4.44 (685/15,414)	0.116	3.23 (545/16,890)	3.40 (88/2,589)	0.689
Loss of taste	4.59 (1,367/29,813)	5.05 (778/15,415)	0.030	3.21 (542/16,884)	4.13 (107/2,589)	0.017

SpO_2_, oxygen saturation.
